# Comparison of hyperbaric ropivacaine with hyperbaric bupivacaine in subarachnoid block

**DOI:** 10.6026/973206300210642

**Published:** 2025-04-30

**Authors:** Anshumali Anshumali

**Affiliations:** 1 Department of Anesthesiology, Critical Care and Pain Management, Shri Ram Murti Smarak Institute of Medical Sciences, Bareilly, Uttar Pradesh, India

**Keywords:** Bupivacaine, hemodynamics, ropivacaine, spinal anesthesia, sensory block

## Abstract

The efficacy and safety of hyperbaric ropivacaine with hyperbaric bupivacaine in spinal anesthesia for lower abdominal surgeries is of interest. Both agents produced reliable sensory and motor blocks; however, ropivacaine exhibited a slower onset, a less extensive sensory block and a faster regression compared to bupivacaine. The duration of the motor block was significantly shorter with ropivacaine. Hemodynamic changes and side effects were similar in both groups. Ropivacaine's favorable recovery profile, including quicker micturition, suggests its potential for daycare surgery. However, the non-availability of a standard densitometer and the need for aseptic preparation of hyperbaric ropivacaine is the limitation.

## Background:

The technique of spinal anesthesia was initially attributed to J. Leonard Corning, a neurologist from New York. During an experiment with cocaine on a dog's spinal nerves, he inadvertently punctured the dura mater, then repeated the process intentionally and named it spinal anesthesia. He proposed its potential application in surgery, deeming the observation noteworthy for documentation [1]. In 1891, Heinrich Irenaeus Quincke formalized lumbar puncture as a straightforward clinical procedure [2]. In 1898, August Bier was the first to employ spinal anesthesia for surgical applications [3]. Bupivacaine, introduced in 1957, was first clinically reported by Dr. Leo Telivuo in 1963, signifying a notable advancement in anesthesia [4]. Spinal anesthesia has gained global popularity due to its benefits, including maintaining patient consciousness, reducing pharmaceutical expenses and facilitating expedited patient turnover. This has emerged as the preferred technique for surgeries involving the lower extremities [5]. Regional anesthetic procedures often offer sufficient pain management, encouraging early mobilization and aiding rehabilitation. The method results in a reduced surgical stress response enhanced cardiac stability, expedited recovery of bowel function and a lower risk of thromboembolism [6]. Numerous anaesthesiologists contend that regional anesthesia yields superior outcomes compared to general anesthesia, which is associated with a higher incidence of problems [7]. Two principal categories of regional anesthesia exist: central neuraxial blockade and peripheral nerve blockade. Central neuraxial blocking is further categorized into epidural and spinal anesthesia. Both procedures entail the application of local anesthetic agents to nerve fibers coming from the spinal cord. Epidural anesthesia involves the injection of the drug into the epidural space, whereas spinal anesthesia entails the administration of the drug into the subarachnoid space [8].

In underdeveloped nations like India, spinal anesthesia is predominantly utilized owing to its affordability, particularly for abdominal, perineal and lower limb surgical procedures. Local anesthetics induce a conduction block of spinal nerves by adhering to nerve fibers within the subarachnoid space [9]. The degree of sympathetic, sensory and motor effects is contingent upon the distribution of the local anesthetic, whilst its removal dictates the duration of these effects. Long-acting local anesthetics are predominantly used in localized anesthesia and analgesia [10]. Ropivacaine, an innovative long-acting local anesthetic, has garnered interest for its superior cardiovascular safety relative to bupivacaine. Research indicates that ropivacaine exhibits a reduced occurrence of clinical cardiac adverse effects compared to bupivacaine. Ropivacaine, analogous to bupivacaine in chemical composition, has been investigated for subarachnoid blocks. Preliminary research has demonstrated its safety and reduced duration of effect compared to bupivacaine, accompanied by a lower incidence of transient neurological symptoms [11]. Recent studies have evaluated ropivacaine versus bupivacaine, determining that ropivacaine is 30-40% less effective and has a shorter duration of action, rendering it appropriate for short to intermediate-length surgeries or outpatient procedures. When identical doses of both anesthetics were evaluated, the onset and degree of sensory blockade were comparable. The length of the sensory block and the extent of the motor block were observed to be reduced with ropivacaine [12, 13]. At present, only isobaric formulations of ropivacaine are available for commercial use. Maintaining the pharmacological stability of hyperbaric solutions for clinical application presents issues. This study aims to evaluate the clinical efficacy of hyperbaric 0.75% ropivacaine (rendered hyperbaric with sterile 25% dextrose) in comparison to commercial hyperbaric 0.5% bupivacaine, using equivalent doses (15 mg) and to determine the viability of ropivacaine as a substitute for bupivacaine in intermediate-duration surgeries under subarachnoid block. Therefore, it is of interest to assess the efficacy of hyperbaric ropivacaine and hyperbaric bupivacaine in subarachnoid block.

## Methodology:

This study involved 100 patients aged 18 to 60 years, scheduled for elective lower abdomen operations and classified as ASA grade I and II. The patients were randomly allocated into two equal groups of 50 individuals each. Group A (Ropivacaine Group) was administered 3 mL of hyperbaric ropivacaine, which was prepared aseptically by combining 2 mL of 0.75% ropivacaine with 1 mL of 25% dextrose, as there was no commercially available formulation available. Group B (Bupivacaine Group) received 3 mL of commercially available hyperbaric 0.5% bupivacaine. A comprehensive pre-anesthetic assessment was conducted, encompassing meticulous history-taking, clinical examination and investigations, including complete blood count, coagulation profile, urinalysis, blood glucose levels, renal function tests, chest X-ray, ECG and screening for Hepatitis B and HIV. The ASA grading was validated during this evaluation. Patients were briefed on the anesthetic procedure and administered 0.25 mg of Alprazolam in tablet form the evening before surgery, along with intramuscular injections of Ranitidine (50 mg) and Glycopyrrolate (0.2 mg) one hour before induction. The utilized materials were typical anesthetic drugs and equipment, including 5 ml syringes, a 25G Quincke spinal needle, antiseptics and sterile instruments. In the operating room, patients were identified, monitored and administered a preload of 10 ml/kg of lactated Ringer's solution. The subarachnoid block was performed under aseptic conditions at the L3-L4 level via the midline approach, with the anesthetic solution administered gradually following confirmation of cerebrospinal fluid flow. Patients were subsequently positioned supinely and continuous monitoring was conducted throughout the procedure. The sensory block was evaluated utilizing the pinprick method at two-minute intervals, recording the duration to reach the T10 level, the maximum dermatomal level attained and the time for two-segment regression. The motor block was assessed using the Modified Bromage Scale, which documented the onset and duration of motor blockade. Hemodynamic parameters, including heart rate, blood pressure, mean arterial pressure, oxygen saturation and electrocardiogram, were assessed every 5 minutes. Hypotension was treated with intravenous ephedrine, while bradycardia was addressed with atropine. Any adverse symptoms such as nausea, vomiting, shivering, respiratory depression, or post-dural puncture headache were documented. In the postoperative period, patients were monitored in the PACU until they had achieved full sensory and motor recovery. Rescue analgesia was administered with intramuscular diclofenac as needed and the time to the first analgesic requirement was recorded. Statistical analysis was performed using SPSS version 22.0. Data were expressed as mean ± SD or frequencies, with appropriate tests such as one-way ANOVA and Mann-Whitney U used based on data distribution. A p-value less than 0.05 were considered statistically significant. The study aimed to evaluate hyperbaric ropivacaine as a potential alternative to hyperbaric bupivacaine for spinal anesthesia in intermediate-duration surgeries, focusing on the onset, duration of sensory and motor block, hemodynamic stability and side effect profile.

## Results:

A total of 100 patients were included in the study, with 50 patients in each group: Group R (Ropivacaine) and Group B (Bupivacaine). Both groups were comparable in terms of age, gender, weight, height and BMI. The mean age was 36.95 ± 11.7 years in Group R and 38.60 ± 12.6 years in Group B (p>0.05). The gender distribution was 70% female and 30% male in Group R, while Group B had 78% female and 22% male patients. The mean BMI was similar between the two groups (p > 0.05), but a significant height difference was observed (p < 0.05). The types of surgeries performed were abdominal hysterectomy, lower segment cesarean section (LSCS), hernia repair and other lower abdominal procedures. There was no statistically significant difference in the type of surgery between the two groups (p > 0.05). The mean duration of surgery was 102.8 ± 10.32 minutes in Group R and 100.92 ± 9.80 minutes in Group B, with no significant difference (p < 0.05). The onset of sensory block was 4.8 ± 2.3 minutes in Group R and 3.5 ± 2.1 minutes in Group B (p>0.05). The onset of motor block was significantly shorter in Group B (6.16 ± 1.24 minutes) than in Group R (9.04 ± 1.20 minutes, p<0.001). The mean highest level of sensory block achieved was T8 in Group R and T7 in Group B. The mean time to peak sensory block was 13.2 ± 2.9 minutes in Group R and 14.6 ± 3.1 minutes in Group B (p=0.021). The mean time to complete motor blockade was 15.8 ± 6.5 minutes in Group R and 12.3 ± 5.9 minutes in Group B (p=0.005) (Table 1, Figure 1 - (see PDF)).

Sensory block regression to S1 was significantly shorter in Group R (154.96 ± 7.57 minutes) compared to Group B (185.20 ± 10.14 minutes, p<0.001). Motor block duration was 131.76 ± 5.61 minutes in Group R and 170.80 ± 8.82 minutes in Group B (p<0.001) (Table 2, Figure 2 (see PDF)). There were no significant differences between the two groups in heart rate, systolic blood pressure (SBP), diastolic blood pressure (DBP), mean arterial pressure (MAP) and oxygen saturation throughout the perioperative period (p > 0.05). Hypotension occurred in 20% of patients in Group R and 28% in Group B (p = 0.241). Bradycardia was observed in 8% of patients in Group R and 12% in Group B (p = 0.487). Backache was reported in 10% of Group R and 16% of Group B (p = 0.311). Post-dural puncture headache (PDPH) occurred in 6% of Group R and 4% of Group B (p=0.587). The mean time to first micturition was 255.37 ± 28.34 minutes in Group R and 340.00 ± 30.31 minutes in Group B, showing a highly significant difference (p = 0.002).

## Discussion:

Ropivacaine, a newer amino-amide local anesthetic, is chemically similar to bupivacaine but 30-40% less potent. It has been studied for spinal anesthesia, demonstrating a shorter duration of action and a lower incidence of transient neurological symptoms (TNS) compared to intrathecal lignocaine. The use of hyperbaric local anesthetics (LA) has become increasingly popular due to their predictable block characteristics [14]. This study aims to compare the effects of hyperbaric ropivacaine with those of hyperbaric bupivacaine in subarachnoid blocks. A total of 100 patients (18-60 years old, ASA grade I and II) undergoing elective lower abdominal surgery were randomly divided into two groups. Group R: Received 3 ml of hyperbaric ropivacaine, prepared by mixing 2 ml of 0.75% ropivacaine with 1 ml of 25% dextrose. Group B: Received 3 ml of commercially available 0.5% hyperbaric bupivacaine. A study by Xu *et al.* [15] with isobaric ropivacaine showed variable block patterns. The addition of glucose was found to enhance its effectiveness, as seen in previous studies on tetracaine and bupivacaine. Our study confirms that 3 mL of 0.5% hyperbaric ropivacaine with 25% dextrose produces a reliable spinal block comparable to that of hyperbaric bupivacaine. The research compares the median effective dose (ED_50_) of hyperbaric and isobaric ropivacaine solutions for knee arthroscopy. The study found that the ED_50_ of hyperbaric ropivacaine (6.55 mg) was lower than that of isobaric ropivacaine (9.71 mg), indicating that adding glucose to ropivacaine to create a hyperbaric solution enhances its anesthetic potency. Several studies have reported conflicting findings regarding the sensory and motor block characteristics of ropivacaine compared to bupivacaine. McNamee *et al.* found that isobaric ropivacaine and bupivacaine had a similar onset and extent of sensory block, but ropivacaine regress faster [16]. Whiteside *et al.* observed that hyperbaric ropivacaine produced a slower onset and shorter duration of sensory and motor block than bupivacaine [17]. Our study found that the onset of sensory block was 4.8±2.3 minutes in Group R and 3.5±2.1 minutes in Group B (p < 0.05) and the onset of motor block was also significantly shorter in Group B (p < 0.001). Regarding the sensory block height, our findings align with those of McDonald *et al.* who reported that equal doses of hyperbaric ropivacaine and bupivacaine achieved similar sensory levels (T7 for bupivacaine and T8 for ropivacaine). However, the mean duration of sensory regression to S1 was significantly shorter in Group R (154.96 ± 7.57 min) than in Group B (185.20 ± 10.14 min). Similarly, the mean motor block duration was 131.76 ± 5.61 minutes in Group R and 170.80 ± 8.82 minutes in Group B (p < 0.05) [18]. Our results showed no significant hemodynamic differences between the groups (p>0.05). Other studies, including those by Whiteside *et al.* [17], support our findings. Side effects were minimal and comparable, with hypotension observed in 20% of patients in Group R and 28% in Group B. The findings of this study align with previous research comparing Ropivacaine and Bupivacaine in spinal anesthesia. Luck *et al.* [19] reported a delayed onset of sensory and motor blocks with Ropivacaine compared to Bupivacaine, a trend reflected in our results (4.8 ± 2.3 min vs. 3.5 ± 2.1 min, p < 0.05 for sensory onset; 9.04 ± 1.20 min vs. 6.16 ± 1.24 min, p < 0.001 for motor onset). Bigat *et al.* [20] highlighted the hemodynamic stability of both agents, with no significant differences in heart rate, blood pressure, or oxygen saturation-findings consistent with our study (p > 0.05). Additionally, the incidence of hypotension and bradycardia was lower with Ropivacaine, reinforcing its safety profile for spinal anesthesia. Carvalho *et al.* [21] emphasized the shorter duration of sensory and motor blockade with Ropivacaine, which was corroborated in our study (sensory regression: 154.96 ± 7.57 min vs. 185.20 ± 10.14 min, p < 0.001; motor block duration: 131.76 ± 5.61 min vs. 170.80 ± 8.82 min, p < 0.001). This faster recovery supports its suitability for procedures requiring early ambulation. Overall, our findings reaffirm that while Bupivacaine provides a longer duration of anesthesia, Ropivacaine offers faster recovery with comparable hemodynamic stability, making it a favorable choice for outpatient and short-duration surgical procedures.

## Conclusion:

Ropivacaine provided effective and reliable spinal anesthesia with a shorter motor block duration but adequate sensory block for moderate-duration surgeries. Its rapid recovery profile and favorable hemodynamic stability make it suitable for day-care surgeries. However, since hyperbaric ropivacaine is not commercially available, its preparation requires careful antiseptic measures. Future studies should focus on further evaluating the role of ropivacaine in ambulatory anesthesia.

## Figures and Tables

**Table 1:** Comparison of onset times of sensory and motor blocks between group R and group B in subarachnoid block (sab)

**Subarachnoid block (SAB)**	**Group R (N=50)**	**Group B (N=50)**	**P value**
Onset of sensory block (min)	4.8±2.3	3.5±2.1	0.004
Onset of motor block (min)	9.04±1.20	6.16±1.24	<0.001

**Table 2:** Comparison of sensory block regression and duration of grade 0 motor blocks between Group R and Group B in subarachnoid block (SAB)

**Subarachnoid block (SAB)**	**Group R (N=50)**	**Group B (N=50)**	**P value**
Sensory block regression into S1 (min)	154.96 ± 7.57	185.20 ± 10.14	<0.001
Duration of grade 0 motor block (min)	131.76 ± 5.61	170.80 ± 8.82	<0.001

## References

[1] Marx G.F (1994). Reg Anesth..

[2] Frederiks J.A, Koehler P.J (1997). J Hist Neurosci..

[3] https://embryo.asu.edu/pages/august-karl-gustav-bier-1861-1949.

[4] https://www.ncbi.nlm.nih.gov/books/NBK532883/.

[5] Olawin A.M, Das J.M (2025). Spinal Anesthesia..

[6] Nordquist D, Halaszynski T.M (2014). Pain Res Treat..

[7] Folino T.B, Mahboobi S.K (2025). Regional Anesthetic Blocks..

[8] Cook T.M, Counsell D (2009). British Journal of Anaesthesia..

[9] https://www.nysora.com/techniques/spinal-anesthesia-2/.

[10] Becker D.E, Reed K.L (2012). Anesth Prog..

[11] Kuthiala G, Chaudhary G (2011). Indian J Anaesth..

[12] Kulkarni K.R (2014). J Anaesthesiol Clin Pharmacol..

[13] Kalbande J.V (2024). Cureus..

[14] Mohta M (2015). J Anaesthesiol Clin Pharmacol..

[15] Xu T (2015). Int J Clin Exp Med..

[16] McNamee D.A (2002). British Journal of Anaesthesia..

[17] Whiteside J.B (2003). British Journal of Anaesthesia..

[18] McDonald S.B (1999). Anesthesiology..

[19] Luck J.F (2008). Br J Anaesth..

[20] Bigat Z (2006). Clin Drug Investig..

[21] Carvalho A.C (2002). Rev Bras Anestesiol..

